# Warming climate extends dryness-controlled areas of terrestrial carbon sequestration

**DOI:** 10.1038/srep05472

**Published:** 2014-07-01

**Authors:** Chuixiang Yi, Suhua Wei, George Hendrey

**Affiliations:** 1 School of Earth and Environmental Sciences, Queens College, City University of New York, New York 11367, USA; 2 The Graduate Center, Program in Earth and Environmental Sciences, City University of New York, New York 10016, USA

## Abstract

At biome-scale, terrestrial carbon uptake is controlled mainly by weather variability. Observational data from a global monitoring network indicate that the sensitivity of terrestrial carbon sequestration to mean annual temperature (*T*) breaks down at a threshold value of 16°C, above which terrestrial CO_2_ fluxes are controlled by dryness rather than temperature. Here we show that since 1948 warming climate has moved the 16°C *T* latitudinal belt poleward. Land surface area with *T* > 16°C and now subject to dryness control rather than temperature as the regulator of carbon uptake has increased by 6% and is expected to increase by at least another 8% by 2050. Most of the land area subjected to this warming is arid or semiarid with ecosystems that are highly vulnerable to drought and land degradation. In areas now dryness-controlled, net carbon uptake is ~27% lower than in areas in which both temperature and dryness (*T* < 16°C) regulate plant productivity. This warming-induced extension of dryness-controlled areas may be triggering a positive feedback accelerating global warming. Continued increases in land area with *T* > 16°C has implications not only for positive feedback on climate change, but also for ecosystem integrity and land cover, particularly for pastoral populations in marginal lands.

Warming climate is altering climate-control mechanisms of terrestrial carbon sequestration^1^,^2^,^3^,^4^. The direct observational evidence^5^ provided by a global network (FLUXNET) of continuous *in situ* measurements of land-atmosphere exchanges of CO_2_, water vapour and energy across biomes and continents, indicates that terrestrial CO_2_ fluxes are: (1) strongly limited by mean annual temperature (*T*) of less than16°C at mid- and high-latitudes; (2) strongly limited by dryness at mid- and low-latitudes; and (3) co-limited by both temperature and dryness around the mid-latitudinal belt (45°N). The sensitivity of terrestrial CO_2_ fluxes to *T* breaks down at ~16°C, a threshold value above which no further increase of CO_2_ uptake with increasing temperature was observed and dryness influence overrules temperature influence. Here, we examine a hypothesis that the threshold-latitudinal belt at which *T* is 16°C is shifting poleward as the Earth's surface warms and hence the areas of dryness-control of terrestrial CO_2_ fluxes (*T* > 16°C) is expanding. We use global land temperature data^6^,^7^ (1948–2012) to test this hypothesis and examine the potential consequences of warming-induced extension of the dryness-controlled area on climate change.

We calculated land area where *T* is higher than or equal to 16°C for each year during the period from 1948 to 2012 using mean monthly surface temperature data (0.5° × 0.5° resolution) from the National Centers for Environmental Prediction-National Center for Atmospheric Research (NCEP/NCAR) reanalysis dataset. We refer to land area with *T* ≥ 16°C as dryness-controlled areas where terrestrial CO_2_ fluxes are limited by dryness and not by temperature^5^. A pronounced increase in the dryness-controlled area occurred following a slight drop before 1976, mirroring the variation with land warming (Fig. 1a). About 90% of the variance in the extension of the dryness-controlled area was accounted for by land warming (R^2^ = 0.90, p < 0.0001, see Fig. 1b).

We assume that net ecosystem-atmosphere exchanges of CO_2_ (NEE) in the areas close to the cold side (*T* < 16°C) of the shifted boundary are controlled by both temperature and dryness^5^, written as *NEE^B^* that is predicted by equation (1) (see Methods Summary). For NEE in the area on the warm side (*T* > 16°C) of the shifted boundary, it is written as *NEE^D^*, that is determined by dryness alone through equation (2). How will increasing *T* affect NEE in area that shifts from control by both *T* and dryness (*T* < 16°C) to control by dryness alone (*T* > 16°C)? We estimated the difference between *NEE^B^* and *NEE^D^* by applying a NEE model (see Methods Summary) that was derived from datasets collected by a worldwide, tower-based, observational network^5^ to the shifted area. The climate data used in the model were *T* and dyness averaged over the period 1948–2010 with 0.5° × 0.5° spatial resolution in the shifted area. Dryness was calculated from the monthly datasets of net incoming short-wave radiation and net long-wave outgoing radiation of the NCEP/NCAR reanalysis^8^ and the gridded monthly terrestrial precipitation datasets^9^. This data-driven estimate indicates that CO_2_ transfer from the atmosphere to the biosphere is reduced by 27% in the shifted area where *T* changed from less than to greater than16°C. Qualitatively, the model prediction reveals a positive feedback mechanism: climate warming extends the dryness-control area, which reduces CO_2_ transfer from the atmosphere to biosphere. Thus, because the atmopheric CO_2_ concentration will increase at a greater rate, the climate will warm at an accelerating rate due to the positive feedback. If the global area under dryness-control (Fig. 1a) continues to increase at only the same rate as occurred over the preceeding half-century, the warming-induced dryness-controlled area will double by 2050.

With climate warming much of Earth's land has been moderately drying since 1976, averged over all land areas, based on annual Palmer Drought Severity Index (PDSI) estimates (Fig. 2a) derived from the monthly self-calibrated PDSI data 0.5° × 0.5° resolution over the spatial range from 60°S to 70°N^10^,^11^. Our analysis finds that the drying trend in the shifted area was strongest and in land areas where *T* is above 16°C was second strongest (Fig. 2b). The land area where *T* > 16°C encompasses low latitudes in the northern hemisphere, most of Africa, Middle and South America, Australia, South- and Southeast Asia (Fig. 3). In these regions, tower-based FLUXNET observations^12^ document that at ground-level these large land areas indeed are drying up and this is confirmed by remote sensing data^13^. This drying is attributed to increased evaporation and evapotranspiration due to warming. If the trend of drying up over the large land area where T > 16°C continues a strong positive feedback on warming is suggested because of reduced CO_2_ transfer from the atmosphere to land (expansion of the brown areas in Fig. 3) via NEE that is limited substantially by water availability (see Fig. 2b in Ref. 5), thus inducing additional warming. In contrast, in the land area where mean annual temperature is below 16°C (green area in Figure 3) a trend toward greater wetness has been observed with climate warming (Fig. 2).

Two large areas between the cold (<16°C, green color in Fig. 3) and warm zones (>16°C, brown color in Fig. 3) have different performances during El Niño/Southern Oscillation (ENSO) events (Fig. 2a, Table 1). In this analysis we included El Niño years with an oceanic Niño index (ONI) greater than +1.0 during the period between 1948 and 2012, and La Niña years with ONI less than −1.0 (Table 1). Half of the El Niño years were consistent with the cold phases (dips of temperature curve) of the cold part of the land (CPL, green area in Fig. 3), while 70% of the La Niña years were consistent with the warm phases (peaks of temperature curve) of the CPL (Table 1). The CPL warm/cold phases appeared to be opposite of the warm/cold phases of the ENSO cycle. However, the global land area followed the warm/cold phases of the ENSO cycle very well. The CPL wet/dry phases appeared different from that of the global land area (Table 1). However, the wet/dry phases of the warm part of the land (WPL) were very consistent with that of the ENSO cycle, i. e. 90% El Niño years were in the dry phases, while 70% La Niña years were in the WPL wet phases (Fig. 2a). We could not find a better relationship of the WPL warm/cold phases with the ENSO cycle. However, if we assume that WPL temperature responses to the ENSO cycle lag by a year, 90% of El Nino years coincided with the WPL warm phases, while 80% of La Niña years coincided with the WPL cold phases. These fascinating coincidences, that became obvious after lagging the data by a year, can be understood at least theoretically in the following way. In the WPL wet phases, a much larger fraction of net radiation is used for evapotranspiration as latent heat and hence potential warming is reduced, while in the WPL dry phases, comparitively less net radiation is used as latent heat, so the temperature is increased. This energy budget adjustment may need about a year to reach equilibrium for about half of Earth's land. Temperature responses to the ENSO cycle of the shifted area (purple color in Fig. 3) were similar to the responses of the total global land area because the temperature of the shifted area is close to the land-average temperature. However, the pattern with 60% of El Niño years being wetter while 30% La Niña years were dryer for the shifted area is coincident with the typical precipitation patterns of the ENSO cycle reported by NOAA^14^.

The shifted areas are transitional zones where not only is the climate-control mechanism of NEE switched as discussed above, but also meteorological conditions are more variable and vegetation is highly vunerable to climate changes and weather extremes. Dominant vegetations in the shifted regions (purple color in Fig. 3) are open shrublands (25%), croplands (22%), grasslands (7%), and desert (13%) (see Supplementary Table 1). Except for the shifted areas in southeastern China (box 4 in Fig. 3) and southeastern United States (box 1 in Fig. 3), most shifted areas are arid and semi-arid land with typical vegetation of open shrublands and grasslands. The annual NEE of these ecosystems is quite sensitive to climate conditions of low precipitation and high evapotranspiration rates^15^,^16^,^17^,^18^.

The locations of shifted areas in the northern hemisphere are coincident with the descending branch of the Hadley cell (HC) and are consequently associated with low precipitation and high evaporation rates^19^. Several lines of evidence indicate that the HC has intensified and expanded poleward over the past three decades as a consequence of climate warming^20^,^21^,^22^ and that the HC expansion in the northern hemisphere is stronger than in southern hemisphere^23^. The contemporaneous poleward shift of both the HC and the WPL (significant since late 1970s) and location of the WPL with respect to the HC (nothern desending branch of the HC), strongly suggests that the WPL migration poleward is driven by global warming. The drying trend of the shifted area (Fig. 2a–b) should be expected to result in vegetation cover shift, with decreased biodiversity and desertification. A line of evidence from remote sensing imagery indicates that drying is accelerating the degradation of vulnerable shrublands in some semiarid Mediterranean area^24^,^25^.

Division of the land into the WPL and CPL by threshold value (16°C) of annual mean temperature based on 64 years (1948–2012) climate data brings new insights into the warming of Earth's surface. The two parts of the land behave almost opposite to each other in the phases of the ENSO cycle and differ in climate control mechanisms of carbon sequestration^5^,^26^,^27^. The WPL has expanded poleward significantly (Fig. 1) and has become dryer (Fig. 2) in the past four decades. The trend of warming-induced drying of the WPL, by reducing NEE thereby reducing withdraw of CO_2_ from the atmosphere, contributes a positive feedback on global warming. Furthermore, as lands are shifted from CPL to WPL becoming more arid and subject to desertification, they also release soil carbon adding additional CO_2_ to the atmosphre. It is estimated that 19–29 Pg of carbon were added to the atmosphere from vegetation and soil carbon pools globally by desertification^28^. The frontal boundary (or the shifted area) of the WPL has been transformed by global warming into more vunerable regions where weather gradients are stronger (Fig. 2), ecosystems are more sensitive to even slight increases in water deficit (Fig. 3)^25^, crop yield is reduced by extreme heat waves^29^, and vegitated land cover and pastroral population are reduced. For instance, in Australia, where wide areas are becoming not suitable for sheep breeding due to reduced precipitation and increased soil salinity. An expansion of the global network^30^ monitoring NEE to target the identified shifted areas would provide data that could improve our ability both to model these regions as they undergo further transitions and to assess the likely impacts on climate as a consequence of altered NEE and increased soil aridity. The present work raises the following two questions: (1) what atmospheric circulation mechanisms support the hypothesis of a year time lag between the WPL temperature response and the ENSO water phases; and (2) is the synergistic poleward expansion of the frontal boundary of the WPL with the HC a long-term or a short-term behavior and what are the consequences of this synergy for global NEE and for the rate of change in atmospheric CO_2_?

## Methods

**Figure 3:**
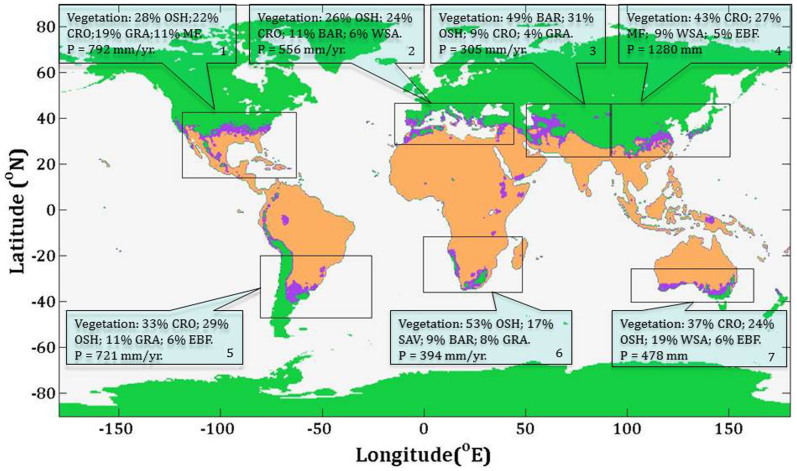
Map of mean annual temperature (1948–2012): below 16°C in light green regions; above 16°C in light red regions, and shift from below 16°C to above 16°C in purple regions. The map was produced based on the NCEP/NCAR ranalysis data^6^,^7^ (http://www.esrl.noaa.gov/psd/data/gridded/data.ncep.reanalysis.derived.pressure.html), and created using Matlab. The information of vegetation distribution, and precipitation (P) are summarized in boxes for the shifted areas (purple color) in each of seven framed regions marked on the map. The vegetation is coded according to the IGBP classification: GRA, grassland; CRO, cropland; MF, mixed forest; OSH, open shrubland; WSA, woody savanna; SAV, savanna; EBF, evergreen broad-leaf forest; and BAR, Barren or sparsely vegetated.

Details of calculating land temperature, precipitation, net radiation, and PDSI are given in the Supplementary Information. Here we summarize the method used to estimate NEE difference induced by the switch of climate control from the CPL to WPL. For the case of the shifted area in the CPL (purple in Fig. 3), its *NEE^B^* is limited by both temperature (*T*) and dryness (*D*) and is estimated using a bivariate-nonlinear regression model 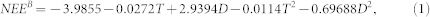
where *D* is defined as *R_n_*/(λ*P*), *R_n_* is mean annual net radiation MJ m^−2^ yr^−1^, *P* is mean annual precipitation mm yr^−1^, and *λ* ( = 2.5 MJ kg^−1^) is the enthalpy of vaporization. For another case of the shifted area in the WPL where *NEE^D^* is limited by dryness along (*T* > 16°C), we use the regression model of *D*-limited group in Ref.^5^, 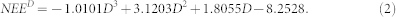
All the regression coefficients in equations (1)–(2) are estimated from the published eddy-covariance data (see supplementary Table S1 in Ref. 5).

## Author Contributions

C.Y. designed the study, conducted data analysis and wrote the manuscript. S.W. performed all calculations and wrote the method part. G.H. contributed to writing the paper and the idea to link the HC extension.

## Supplementary Material

Supplementary Information SUPPLEMENTARY INFO

## Figures and Tables

**Figure 1:**
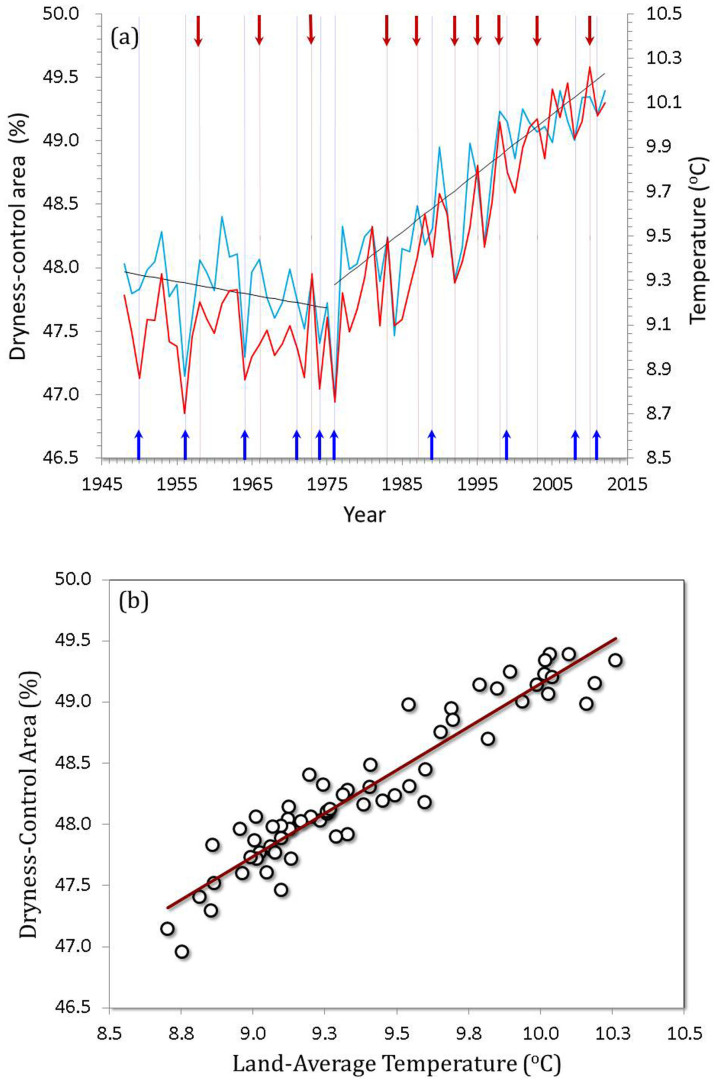
Relationship between dryness-control area (%)and land temperature (°C) (1948–2012): (a) the evolution of dryness-control area (blue line) and land average temperature (red line); and (b) correlation between annual dryness-control area and annual land-average temperature (R^2^ = 0.90, P < 0.0001). The dryness-control area refers to the total area of regions where mean annual temperature was higher than or equal to 16°C and terrestrial CO_2_ fluxes are controlled by dryness rather than temperature based on the direct observational evidence provided by a global monitoring network^5^. The annual dryness-control area and annual land surface temperature during the period from 1948 to 2012 were derived from mean monthly temperature data at surface (0.5° × 0.5° resolution) from the National Centers for Environmental Prediction-National Center for Atmospheric Research (NCEP/NCAR) reanalysis data set^6^,^7^. The black lines indicate the trends of dryness-control area that was similar to that of land-average temperature (omitted): a slight drop between 1948–1975 and then a striking increase during 1976–2012. The striking increase in temperature is a direct result of increased greenhouse gases in the atmosphere^31^. The red arrows in (a) indicate El Niño years with oceanic Niño index (ONI) greater than +1.0, while blue arrows in (a) indicate La Nina with ONI less than −1.0 (http://www.cpc.ncep.noaa.gov/products/analysis_monitoring/ensostuff/ensoyears.shtml). 70% El Niño years were consistent with warmer years, while 80% La Nina years were consistent with cooler years.

**Figure 2:**
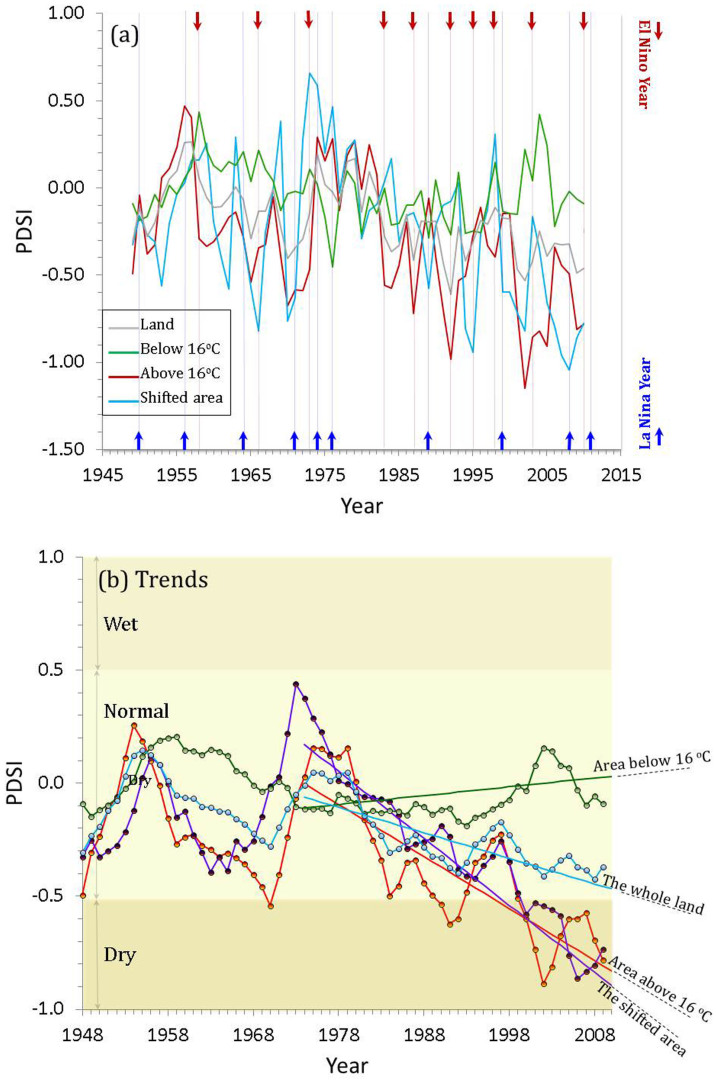
The Palmer Drought Severity Index (PDSI). (a) Links between PDSI and ENSO events.The red curve shows PDSI for the area with temperature above 16°C; the green curve for the area with temperature below 16°C; the grey curve for the whole area of the land; and the blue curve for the shifted area from below 16°C to above 16°C during 1948–2012. The red arrows indicate El Niño years with oceanic Niño index (ONI) greater than +1.0, while blue arrows indicate La Niña with ONI less than −1.0 (http://www.cpc.ncep.noaa.gov/products/analysis_monitoring/ensostuff/ensoyears.shtml). (b) The trends of PDSI. The filled circles are five-year moving average of the PDSI data shown in (a). Mean annual land surface temperature during the period from 1948 to 2012 was derived from mean monthly temperature data at surface (0.5° × 0.5° resolution) from the National Centers for Environmental Prediction-National Center for Atmospheric Research (NCEP/NCAR) reanalysis data set^6^,^7^. Annual PDSI data were derived from the monthly self-calibrated PDSI data (0.5° × 0.5° resolution, spatial range from 60°S to 70°N, http://www.cgd.ucar.edu/cas/catalog/climind/pdsi.html)^11^,^12^. Drought classification by PDSI are: [−0.49, +0.49] → normal; [−0.5, −0.99] → incipient dry spell; [−1.0, −1.99] → mild drought; [−2.0, −2.99] → moderate drought; [−3.0, −3.99] → severe drought^10^. The PDSI behaviours to the ENSO events were different between: (1) the area above 16°C (red curve in (a)), 90% El Nino years were dryer, while 70% La Nina years were wetter; and (2) the area below 16°C (green curve (a)), 50% El Nino years were wetter, while 40% La Nina years were dryer (see Table 1).

**Table 1:** Temperature, PDSI, and ENSO events from different areas classified in Figure 3

	Properties	10 El Nino years*	10 La Nina years+
Global Land Area	T (°C)	70% warmer	80% cooler
	PDSI	30% dryer	40% wetter
Land Area Above 16°C	T (°C)	Not clear but taking 1 year lag 90% warmer	Not clear but taking 1 year lag 80% cooler
	PDSI	90% dryer	70% wetter
Land Area Below 16°C	T (°C)	50% cooler	70% warmer
	PDSI	50% wetter	40% dryer
The shifted area	T (°C)	70% warmer	80% cooler
	PDSI	60% wetter	30% dryer

*10 El Nino years include: 1957–1958, 1965–1966, 1972–1973, 1982–1983, 1986–1987, 1991–1992, 1994–1995, 1997–1998, 2002–2003, 2009–2010.

+10 La Nina years include: 1950–1951, 1955–1956, 1964–1965, 1970–1971, 1973–1974, 1975–1976, 1988–1989, 1998–1999, 2007–2008, 2010–2011.

## References

[b1] Canadell J. G. *et al.* Contributions to accelerating atmospheric CO_2_ growth from economic activity, carbon intensity, and efficiency of natural sinks. Proc Nat Acad Sci USA 104, 18866–18870 (2007). 17962418 10.1073/pnas.0702737104 PMC2141868

[b2] Cox P. M., Pearson D., Booth B. B., Friedlingstein P., Huntingford C., Jones C. D. & Luke C. M. Sensitivity of tropical carbon to climate change constrained by carbon dioxide variability. Nature 494, 341–344 (2013). 23389447 10.1038/nature11882

[b3] Zhao M. & Running S. W. Drought-induced reduction in global terrestrial net primary production from 2000 through 2009. Science 329, 940–943(2010). 20724633 10.1126/science.1192666

[b4] Ciais Ph. *et al.* Europe-wide reduction in primary productivity caused by the heat and drought in 2003. Nature 437. 529–533 (2005). 16177786 10.1038/nature03972

[b5] Yi C. *et al.* Climate control of terrestrial carbon exchange across biomes and continents. Environ. Res. Lett. 5, 1748–9326 (2010).

[b6] Kalnay E. *et al.* The NCEP/NCAR 40-year reanalysis project. Bull. Amer. Meteor. Soc. 77, 437–470 (1996).

[b7] Fan Y. & van den Dool H. A global monthly land surface air temperature analysis for 1948-present. J. Geophys. Res. 113, D01103, 10.1029/2007JD008470 (2008).

[b8] Shi Q. & Liang S. Characterizing the surface radiation budget over the tibetan plateau with ground-measured, reanalysis, and remote sensing datasets. Part 1: Methodology. J. Geophys. Res. 118, 8921–8934 (2013).

[b9] Chen M., Xie P., Janowiak J. E. & Arkin P. A. Global land precipitation: a 50-yr monthly analysis based on gauge observations. J. Hydrometeor. 3, 249–266, (2002).

[b10] Dai A. Characteristics and trends in various forms of the Palmer Drought Severity Index (PDSI) during 1900–2008. J. Geophys. Res. 116, D12115 (2011a) http://www.cgd.ucar.edu/cas/catalog/climind/pdsi.html.

[b11] Dai A. Drought under global warming: A review. Wiley Interdisciplinary Reviews: Climate Change 2, 45–65 (2011b).

[b12] Mu Q., Zhao M. & Running S. W. Improvements to a MODIS global terrestrial evapotranspiration algorithm. Remote Sens. Environ. 115, 1781–1800 (2011).

[b13] Jung M. *et al.* Recent decline in the global land evapotranspiration trend due to limited moisture supply. Nature 467, 951–954 (2010). 20935626 10.1038/nature09396

[b14] Yan H. *et al.* Diagnostic analysis of interannual variation of global land evapotranspiration over 1982–2011: Assessing the impact of ENSO. J. Geophys. Res. 118, 8969-8983 (2013).(http://www.cpc.ncep.noaa.gov/products/precip/CWlink/MJO/enso.shtml).

[b15] Prieto P., Peñuelas J., Ogaya R. & Estiarte M. Precipitation-dependent Flowering of *Globularia alypum* and *Erica multiflora* in Mediterranean Shrubland Under Experimental Drought and Warming, and its Inter-annual Variability. Ann. Bot. 102, 275–285 (2008). 18565983 10.1093/aob/mcn090 PMC2712364

[b16] Noy-Meir I. Desert ecosystems. I. Environment and producers. Annu. Rev. Ecol. Syst. 4, 25–52 (1973).

[b17] Warner T. T. Desert Meteorology 595 pp., Cambridge University Press, New York. (2004).

[b18] Yi C. *et al.* Climate extremes and grassland potential productivity. Environ. Res. Lett. 7, 035703 (6pp) 10.1088/1748-9326/7/3/035703 (2012).

[b19] Kang S. & Lu J. Expansion of the Hadley cell under global warming: winter versus summer. J. Clim. 25, 8387–8393 (2012).

[b20] Hu Y. & Fu Q. Observed poleward expansion of the Hadley circulation since 1979. Atmos. Chem. Phys. 7, 5229–5236 (2007).

[b21] Lu J., Vecchi G. & Reichler T. Expansion of the Hadley cell under global warming. Geophys. Res. Lett. 34, L06805, 10.1029/2006GL028443 (2007).

[b22] Li W., Li L., Ting M. & Liu Y. Intensification of Northern Hemisphere Near-Surface Subtropical Highs in a Warming Climate. Nature Geosci. 5, 830–834 (2012).

[b23] Nguyen H. *et al.* The Hadley Circulation in Reanalyses: Climatology, Variability, and Change. J. Clim. 26, 3357–3376 (2013).

[b24] Vicente-Serrano S. M., Zouber A., Lasanta T. & Pueyo Y. Dryness is accelerating degradation of vulnerable shrublands in semiarid Mediterranean environments. Ecol Monogr 82, 407–428 (2012).

[b25] Dorman M., Svoray T. & Perevolotsky A. Homogenization in forest performance across an environmental gradient – The interplay between rainfall and topographic aspect. Forest Ecol. Manage. 310, 256–266 (2013).

[b26] Graven H. D. *et al.* Enhanced seasonal exchange of CO_2_ by northern ecosystems since 1960. Science 341, 1085–1089 (2013). 23929948 10.1126/science.1239207

[b27] Pen S. *et al.* Asymmetric effects of daytime and night-time warming on Norther Hemisphere vegetation. Nature 501, 88–92 (2013). 24005415 10.1038/nature12434

[b28] Lal R. Carbon sequestration in dryland ecosystems. Environ. Manage. 33, 528–544 (2004). 15453406 10.1007/s00267-003-9110-9

[b29] Lobell D. B. & Gourdji S. M. The influence of climate change on global crop productivity. Plant Physiol. 160, 1686–1697 (2012). 23054565 10.1104/pp.112.208298 PMC3510102

[b30] Baldocchi D. *et al.* FLUXNET: A new tool to study the temporal and spatial variability of ecosystem-scale carbon dioxide, water vapor, and energy flux densities. Bull. Am. Meteorol. Soc. 82, 2415–2434 (2001).

[b31] Rohde R. *et al.* A New Estimate of the Average Earth Surface Land Temperature Spanning 1753 to 2011. Geoinfor Geostat: An Overview 1, 1. 10.4172/gigs.1000101(2013).

