# Supplementary material for: Warming climate extends dryness-controlled areas of terrestrial carbon sequestration
Source: Sci Rep. 2014 Jul 1;4:5472. doi: 10.1038/srep05472 (PMC4076677; doi:10.1038/srep05472)
Supplement: Supplementary Information — SUPPLEMENTARY INFO [file srep05472-s1.pdf]

Supplementary Materials

Warming climate extends dryness-controlled areas of terrestrial carbon sequestration

Chuixiang Yi, Suhua Wei, George Hendrey

[1]School of Earth and Environmental Sciences, Queens College, City University of New York, New York 11367, USA.

Methods

Temperature. Annual mean temperature was calculated from mean monthly temperature data at surface (0.5^o x 0.5^o resolution) from the National Centers for Environmental Prediction-National Center for Atmospheric Research (NCEP/NCAR) reanalysis data set[6-7]. Monthly land surface temperature was averaged over each grid cell in each year to get annual mean temperature from year 1948 to 2012 (http://www.esrl.noaa.gov/psd/data/gridded/data.ncep.reanalysis.derived.pressure.html).

Precipitation. Annual precipitation over each grid cell (0.5^o x 0.5^o resolution) was summed up for each year from the monthly precipitation data sets with spatial resolution 0.5^o x0.5^o covering the period of Jan 1948 to Dec 2010[10] (http://www.esrl.noaa.gov/psd/data/gridded/data.precl.html). The calculated annual precipitation data were used for dryness calculation.

Land Cover. The land cover classes were determined by MODIS land cover product MOD12C1 dataset. The land cover types were classified under IGBP global vegetation classification scheme. The resolution of original dataset format was 0.05^{o}x0.05^o. We resampled the data sets into 0.5^{o}x0.5^o resolution using nearest neighbor interpolation, which was processed by Matlab Mapping toolbox. Land Cover data was used to define the vegetation types at boundary regions.

Radiation. Monthly radiation data sets were calculated from NCEP reanalysis net shortwave radiation and net longwave radiation covering period of 1948 to 2010. Net radiation is the balance of net incoming radiation (short wave radiation) and net outgoing radiation (long wave radiation).The radiation reanalysis data originally used a resolution of 1.875^o x1.915^o [8-9]. We resampled the data into 0.5^{o}x0.5^o resolution using nearest neighbor interpolation, which was performed by Matlab mapping tool box. (http://140.172.38.100/psd/thredds/catalog/Datasets/ncep.reanalysis.derived/surface_gauss/catalog.html).Radiation data sets were used as the input data to calculate dryness.

Dryness. Dryness index was defined as: $Dryness=R_n/(L*P)$, where R_n (MJ m^{-2} yr^{-1}) and P (mm yr^{-1}) are annual mean net radiation and precipitation for each grid cell respectively, and L=2.5 (MJ kg^{-1}) is the enthalpy of vaporization. We excluded a

method to calculate the dryness index on a grid cell because annual precipitation for some grid cells was so low that it would cause the dryness index to be infinite. All desert areas were excluded in dryness calculations.

PDSI. The Palmer Drought Severity Index (PDSI) has been widely used to quantify long-term changes of surface moisture conditions[11]. PDSI is a standardized measurement, ranging from -10 (dry) to +10 (wet) that allows comparisons across space and time[12]. The PDSI data sets used in this analysis cover 60S-77.5N with resolution 2.5^ox2.5^o. We resampled the data into 0.5^ox0.5^o resolution using nearest neighbor interpolation, which was completed by Matlab mapping toolbox. (www.cgd.ucar.edu/cas/catalog/climind/pdsi.html)

Area-weighted approach. We performed an area-weighted mean within a latitude grid box from data given on a regular latitude-longitude grid (0.5^ox0.5^o). Spherical triangle in area calculation method is applied in order to compensate for the meridian convergence toward higher latitudes. The method treated the earth as a spherical ball with a radius of 6371km. Each gridded pixel is broken into its upper left triangle and lower right triangle. Each spherical triangle is calculated by the lat/lons of the pixel using the formula of spherical triangle area calculation. All the area-weighted calculations in this study were performed in this way.

Temporally, a 5-year moving average span was applied to PDSI data shown in Fig. 2b, by using a lowpass filter with filter coefficients equal to reciprocal of the span. Seven sub-regions of the shifted area (purple areas in seven boxes in Fig.3) are arbitrarily assigned to study the features of climate and vegetation. The starting and ending latitudes and longitudes are provided as following:

Purple area in framed boxes in Fig.3	latitudes	longitudes
Box 1	15N-45N	120W-65W
Box 2	22.5N-45N	30W-45E
Box 3	22.5N-45N	50E-90E
Box 4	22.5N-45N	90E-150E
Box 5	30S-60S	90W-30W
Box 6	15S-45S	0E-30E
Box 7	15S-45S	105E-165.5E

Supplementary Table S1. Vegetation distribution (%) in the shifted areas marked in Figure 3.

Vegetation	Region 1	Region 2	Region 3	Region 4	Region 5	Region 6	Region 7	Whole shifted area
ENF	1	1	0	1	0	0	0	1
EBF	1	1	0	5	6	1	6	6
DBF	5	0	0	0	2	0	0	1
MF	11	5	2	27	0	0	0	6
CSH	1	1	0	0	4	0	0	1
OSH	28	26	31	0	29	53	24	25
WSA	6	6	0	9	2	2	19	6
SAV	0	3	0	0	2	17	2	3
GRA	19	2	4	1	11	8	0	7
CRO	22	24	9	43	33	2	37	22
URB	2	2	1	4	1	1	0	2
BAR	1	11	49	2	3	9	0	13

The vegetation is coded according to the IGBP classification: ENF, evergreen needle-leaf forest; EBF, evergreen broad-leaf forest; DBF, deciduous broad-leaf forest; MF, mixed forest; CSH, Closed shrublands; OSH, open shrubland; WSA, Woody savannas; SAV, Savannas; GRA, grassland; CRO, cropland; URB, Urban and built-up; and BAR, barren or sparsely vegetated.

References

1. Canadell, J. G., et al. Contributions to accelerating atmospheric CO2 growth from economic activity, carbon intensity, and efficiency of natural sinks. *Proc Nat Acad Sci USA* **104**,18866–18870 (2007).
2. Cox, P. M., Pearson, D., Booth, B. B., Friedlingstein, P., Huntingford, C., Jones, C. D., Luke, C. M. Sensitivity of tropical carbon to climate change constrained by carbon dioxide variability. *Nature* **494**, 341-344, (2013).
3. Zhao, M., Running, S. W. Drought-induced reduction in global terrestrial net primary production from 2000 through 2009. *Science* **329**, 940-943(2010).
4. Ciais, Ph. et al. Europe-wide reduction in primary productivity caused by the heat and drought in 2003. *Nature* **437**. 529-533 (2005).
5. Yi, C., et al. Climate control of terrestrial carbon exchange across biomes and continents. *Environ. Res. Lett.* **5**, 1748-9326 (2010).
6. Kalnay, E., et al. The NCEP/NCAR 40-year reanalysis project. *Bull. Amer. Meteor. Soc.* **77**, 437-470, 1996.
7. Fan, Y., van den Dool, H. A global monthly land surface air temperature analysis for 1948-present. *J. Geophys. Res.* **113**, D01103, doi:10.1029/2007JD008470 (2008).
8. Shi, Q., Liang, S. Characterizing the surface radiation budget over the tibetan plateau with ground-measured, reanalysis, and remote sensing datasets. Part 1: Methodology. *J. Geophys. Res.* **118**, 8921-8934 (2013).
9. Chen, M., Xie, P., Janowiak, J. E., and Arkin, P. A. Global land precipitation: a 50-yr monthly analysis based on gauge observations. *J. Hydrometeor.* **3**, 249-266, 2002.

10. Dai, A. Characteristics and trends in various forms of the Palmer Drought Severity Index (PDSI) during 1900-2008. *J. Geophys. Res.* **116**, D12115 (2011a). http://www.cgd.ucar.edu/cas/catalog/climind/pdsi.html
11. Dai, A. Drought under global warming: A review. *Wiley Interdisciplinary Reviews: Climate Change* **2**, 45-65 (2011b).
12. Mu, Q., Zhao, M., and Running, S. W. Improvements to a MODIS global terrestrial evapotranspiration algorithm. *Remote Sens. Environ.* **115**, 1781–1800 (2011).
13. Jung, M., et al. Recent decline in the global land evapotranspiration trend due to limited moisture supply. *Nature* **467**, 951-954 (2010).
14. Yan, H., et al. Diagnostic analysis of interannual variation of global land evapotranspiration over 1982–2011: Assessing the impact of ENSO. *J. Geophys. Res.* **118**, 8969-8983 (2013). (http://www.cpc.ncep.noaa.gov/products/precip/CWlink/MJO/enso.shtml)
15. Prieto, P., Peñuelas, J., Ogaya, R., and Estiarte, M. Precipitation-dependent Flowering of *Globularia alypum* and *Erica multiflora* in Mediterranean Shrubland Under Experimental Drought and Warming, and its Inter-annual Variability. *Ann. Bot.* **102**: 275–285 (2008).
16. Noy-Meir, I. Desert ecosystems. I. Environment and producers. *Annu. Rev. Ecol. Syst.* **4**, 25-52 (1973).
17. Warner, T. T. *Desert Meteorology*, 595 pp., Cambridge University Press, New York. (2004).
18. Yi, C. et al. Climate extremes and grassland potential productivity. *Environ. Res. Lett.* **7**, 035703 (6pp) doi:10.1088/1748-9326/7/3/035703 (2012).
19. Kang, S., Lu, J. Expansion of the Hadley cell under global warming: winter versus summer. *J. Clim.* **25**, 8387-8393 (2012).
20. Hu, Y., Fu, Q. Observed poleward expansion of the Hadley circulation since 1979. *Atmos. Chem. Phys.* **7**, 5229-5236 (2007).
21. Lu, J., Vecchi, G., Reichler, T. Expansion of the Hadley cell under global warming. *Geophys. Res. Lett.*, **34**, L06805, doi:10.1029/2006GL028443 (2007).
22. Li, W., Li L., Ting M., Liu Y. Intensification of Northern Hemisphere Near-Surface Subtropical Highs in a Warming Climate. *Nature Geosci.* **5**, 830-834 (2012).
23. Nguyen, H., et al. The Hadley Circulation in Reanalyses: Climatology, Variability, and Change. *J. Clim.* **26**, 3357–3376 (2013).
24. Vicente-Serrano, S. M., Zouber, A., Lasanta, T., Pueyo, Y. Dryness is accelerating degradation of vulnerable shrublands in semiarid Mediterranean environments. *Ecol Monogr* **82**, 407–428 (2012).
25. Dorman, M., Svoray T., Perevolotsky A. Homogenization in forest performance across an environmental gradient – The interplay between rainfall and topographic aspect. *Forest Ecol. Manage.* **310**, 256-266 (2013)
26. Graven, H. D. et al. Enhanced seasonal exchange of CO2 by northern ecosystems since 1960. *Science* **341**, 1085-1089 (2013).
27. Pen, S. et al. Asymmetric effects of daytime and night-time warming on Norther Hemisphere vegetation. *Nature* **501**, 88-92 (2013).
28. Lal, R. Carbon sequestration in dryland ecosystems. *Environ. Manage.* 33, 528-544 (2004).
29. Lobell, D. B., Gourdji, S.M. The influence of climate change on global crop productivity. *Plant Physiol.* **160**, 1686-1697 (2012).

30. Baldocchi, D., et al. FLUXNET: A new tool to study the temporal and spatial variability of ecosystem-scale carbon dioxide, water vapor, and energy flux densities. *Bull. Am. Meteorol. Soc.* 82, 2415-2434 (2001).
31. Rohde, R. et al. A New Estimate of the Average Earth Surface Land Temperature Spanning 1753 to 2011. *Geoinfor Geostat: An Overview* **1**,1. doi:10.4172/gigs.1000101(2013).
32. Delworth, T.D. et al. GFDL's CM2 global coupled climate models-Part I: Formulation and simulation characteristics. *J. Clim.* **19**, 643-674 (2006).
33. Donner, L. J. et al. The Dynamical Core, Physical Parameterizations, and Basic Simulation Characteristics of the Atmospheric Component of the GFDL Global Coupled Model CM3. *J. Clim.* **24**, 3484-3518 (2011).
